# Medical Education Learning Specialists in the Age of Artificial Intelligence

**DOI:** 10.7759/cureus.88439

**Published:** 2025-07-21

**Authors:** Helen Hu, Dechantria D Wallace, Beatrice Boateng

**Affiliations:** 1 Center for Excellence in Teaching and Learning, University of Arkansas Pulaski Technical College, North Little Rock, USA; 2 Educational and Student Success Center, University of Arkansas for Medical Sciences, Little Rock, USA; 3 College of Medicine, University of Arkansas for Medical Sciences, Little Rock, USA

**Keywords:** academic support, artificial intelligence, learning specialists, medical education, student success

## Abstract

Introduction

Generative Artificial Intelligence (AI) is transforming healthcare education by offering innovative solutions to enhance students' learning and skill development. Medical education learning specialists often seek effective strategies to support students in mastering content and improving study habits. This study aimed to investigate learning specialists’ perspectives on the impact of AI on their roles and interactions with healthcare education students.

Methods

The researchers conducted a national online survey with the Medical Education Learning Specialists (MELS) Special Interest Group, a community of professionals providing academic support to medical and health professions students. Sixty-one learning specialists from 29 states of the United States participated in the survey.

Results

Key findings reveal that, despite advances in generative AI, learning specialists agree that their roles in supporting students will remain valuable and irreplaceable. However, they emphasized the need for targeted training to effectively integrate AI into academic support services. Other findings suggest that medical education learning specialists see the significant promise of AI in improving personalization in medical education and student learning experiences.

Conclusion

As healthcare education evolves, the role of generative AI in enhancing personalized learning and efficient study strategies to address the unique needs of students will continue to grow. AI tools can equip learning specialists with the assistance needed to provide targeted support to students.

## Introduction

Education in the healthcare profession is an incredibly challenging field for students and faculty members. Medical education learning specialists often support students challenged by the volume of content in the field and use an individualized teaching approach to support students’ study skills to enhance academic performance. Learning specialists engage with medical students, residents, and other healthcare education students with academic support. Their activities include 1) coordinating programs to support student success, retention, and individualized academic assistance; 2) working with students to identify their learning needs, strengths, and weaknesses, as well as developing academic learning plans; 3) providing sessions on topics like time management, test preparation, and study strategies; 4) supporting students in preparing for professional licensing exams, e.g., the United States Medical Licensing Examination (USMLE); and 5) developing and improving peer tutoring programs [1,2].

Generative artificial intelligence (AI) is defined as computational models that can generate seemingly new, high-quality text, images, and other content based on the data they were trained on [3,4]. Google Gemini (Alphabet, Inc., Mountain View, USA), ChatGPT (OpenAI, Inc., San Francisco, USA), and Microsoft Copilot (Microsoft Corporation, Redmond, USA) are examples of generative AI tools or applications. ChatGPT 4.0 passed both written dental licensing examinations from the US and UK [5]. It also passed the medical licensing examinations in many cases, outperforming the average scores of human medical students in some cases [6].

The emergence of generative AI has ushered in new possibilities for designing and delivering effective learning experiences in various settings. AI can provide personalized learning experiences [7,8], such as in simulation for medical knowledge and skill acquisition [9]. Furthermore, AI can be conducive to coaching [10], such as using a chatbot as a teaching assistant to answer medical students’ questions [11]. ChatGPT can also be used to create personalized study schedules [12] or as a question bank [13] to help students prepare for USMLE Step exams. As a result of the development and integration of AI, one starts to question the significance of the position of medical education learning specialists.

While most studies report positive student perceptions and improved engagement [14] with AI integration, learners expressed frustration with the chatbots' lack of emotional intelligence or empathy, dehumanization of patient care [8], and the technical challenges of limited, nuanced feedback on complex assignments, and privacy and security concerns. Most studies about AI tools have focused on trainees’ knowledge, attitudes, and skills. These studies found fundamental knowledge gaps about how to utilize AI tools [15-17] and students’ concerns about job security [18,19]. However, limited studies exist on learning specialists' perceptions of their roles and whether they have concerns about job security as generative AI tools become prevalent in healthcare education.

The rapid advancements in generative artificial intelligence (AI) technology have changed the landscape of education, including the arena for learning specialists in healthcare education. With the development and integration of AI, people have started to wonder if it is still necessary to keep these positions. Thus, the authors of this article argue that in an age where artificial intelligence (AI) is rapidly transforming the educational landscape, the role of medical education learning specialists has never been more critical. This article explores the evolving responsibilities and opportunities for learning specialists amidst the rise of AI technologies and how AI can be harnessed by learning specialists to augment the learning experience, personalize education, and empower healthcare education students to achieve academic excellence.

## Materials and methods

This study aims to investigate medical education learning specialists’ perspectives on the impact of AI on their work and interactions with healthcare education students. The researchers received Institutional Review Board (IRB) Exempt approval (FWA00001119) from the University of Arkansas for Medical Sciences, at the time of the study, and permission from the leadership of the Medical Education Learning Specialist (MELS) Special Interest Group, a community of professionals providing academic support to medical and other healthcare education students, to send a survey through the organization’s listserv while recruiting participants.

Participants

Study participants are members of the MELS Special Interest Group. A total of 61 (out of 900 registered members in 2024) learning specialists from 29 U.S. states volunteered to complete the survey, which resulted in a response rate of 6.8% for the online survey. Most respondents had an education at either the master's (n=33, 54.1%) or Ph.D. (n=26, 42.6%) level.

Instrument

A two-part online survey (see Appendices) was created in Research Electronic Data Capture (REDCap, Vanderbilt University, Nashville, USA), a secure, web-based application for building and managing online surveys and databases. Part A gathered learning specialists' perspectives on the integration and impact of AI in medical education, focusing on both the opportunities and challenges it presents. Part B addressed the concerns and perceptions about job security among medical education professionals in the context of AI advancements. The survey included items rated on a 5-point Likert scale (1=Strongly Agree, 2=Agree, 3=Neutral, 4=Disagree, 5=Strongly Disagree) and an item for open-ended text responses. In addition to demographic items including a) length of time working with medical students, b) time in current position, c) technology proficiency, d) degree, and e) state of medical school, participants were asked if they had adopted any AI tools and were offered a textbox to provide any additional narrative comments regarding their experience with the tools they adopted (see Appendices). No names or any personal identifiers of the participants were collected.

The online survey link was sent through the MELS organization listserv on October 23, 2024, before the 2024 MELS conference in November 2024. A reminder was sent on October 30, 2024, which was one week before the conference. The findings were presented during a round-table discussion at the conference.

## Results

A total of 61 learning specialists from 29 U. S. states completed the survey. Most respondents either held a master's degree (n=33, 54.1%) or a Ph.D. (n=26, 42.6%). A majority also indicated that their technology proficiency was either intermediate, meaning they were comfortable using common software tools (n=23, 37.7%), or advanced, meaning they were able to troubleshoot and use advanced software features (n=30, 49%). Survey responses were entered into R (The R Project for Statistical Computing, Vienna, Austria) and cleaned before analysis. To investigate the relationships between variables, Chi-square analyses were conducted. It was found that there is neither a statistically significant association between technology proficiency and the confidence that their expertise and skills will remain valuable (p = 0.8415) nor between technology proficiency and the belief that AI will assist rather than replace educators (p = 0.866). Additionally, through Chi-square, it was found that there is neither a statistically significant association between AI tool adoption and technology proficiency (p = 0.728) nor between AI tool adoption and the belief that AI will replace tasks performed by learning specialists (p = 0.288). This article only explores participants' perspectives on AI at a single time point; quantitative data were only reported using descriptive statistics, such as frequencies, percentages, means, and standard deviation. Qualitative data from participants’ narrative responses to the survey or conference discussions were analyzed using content analysis.

Medical education learning specialists' perspectives

A majority of respondents (n=57, 93%) agreed or strongly agreed that medical education learning specialists will continue to play a critical role in medical education despite advances in AI. Please see Table 1 for the frequency count of participants' responses regarding their perspectives.

**Table 1:** Frequency for perspectives on the integration and impact of AI in medical education (N=61). Response options (1=Strongly Agree, 2= Agree, 3= Neutral,  4=Disagree, 5=Strongly Disagree). Data are presented as numbers (frequency counts and % in 61 participants).

Question	Strongly Agree	%	Agree	%	Neutral	%	Disagree	%	Strongly Disagree	%
AI technology can significantly improve the personalization of medical education.	5	8%	22	36%	25	41%	9	15%	0	0%
AI-driven analytics are more effective than traditional methods in identifying areas where medical students need improvement.	2	3%	10	16%	30	49%	18	30%	1	2%
Medical education learning specialists need to develop new skills to effectively incorporate AI into their practices.	24	39%	31	51%	4	7%	2	3%	0	0%
Medical education learning specialists will continue to play a critical role in medical education despite advances in AI.	46	75%	11	18%	2	3%	1	2%	1	2%
The adoption of AI in medical education will widen the digital divide between different institutions and students.	5	8%	21	34%	25	41%	9	15%	1	2%
The integration of AI tools in medical education enhances the learning experience for students.	11	18%	26	43%	22	36%	2	3%	0	0%

**Figure 1:**
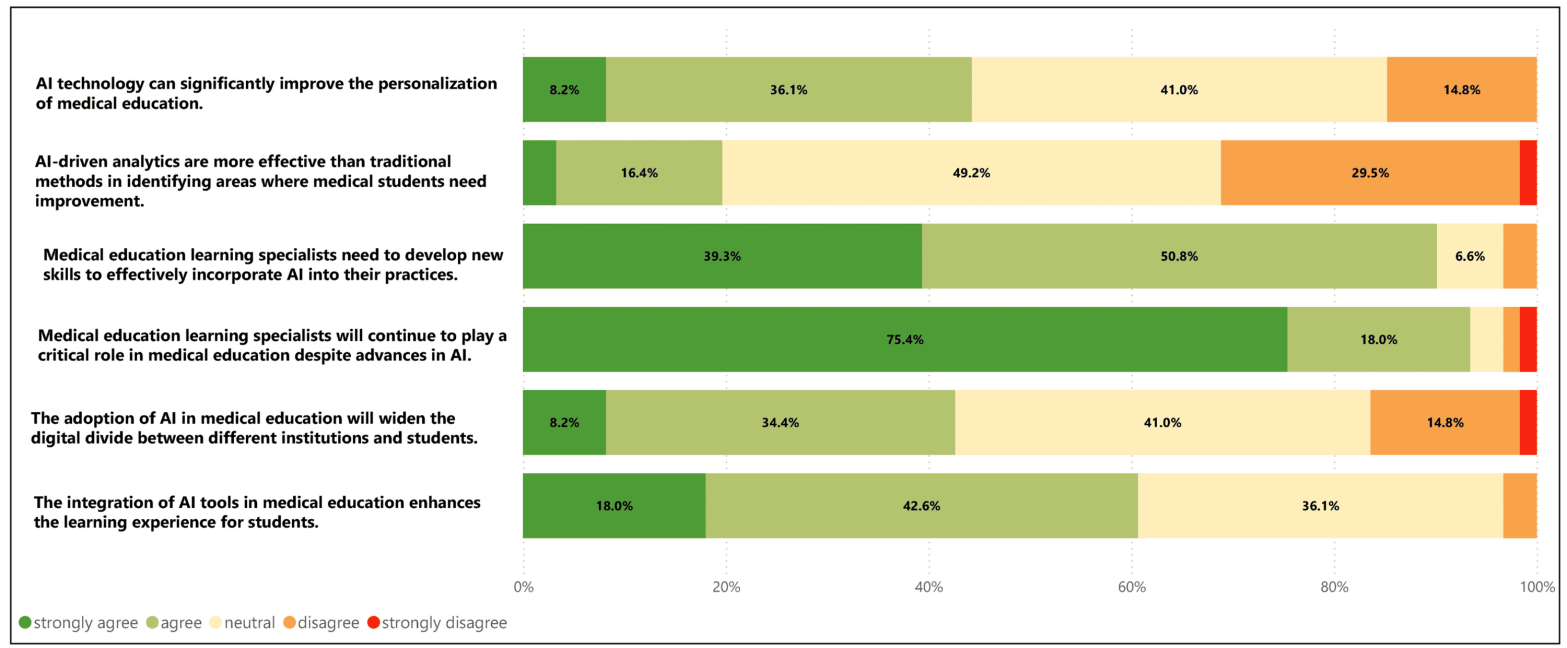
Medical education learning specialists' perspectives on the integration and impact of AI in medical education (N=61).

About 45% (n=27) indicated that it could significantly improve the personalization of medical education and enhance student learning experiences (n=37, 60%). Even though AI could enhance the learning experience, only 20% (n=12) agreed or strongly agreed that AI-driven analytics are more effective than traditional methods in identifying areas where medical students need improvement. Most were unsure (n=30, 49%) or disagreed (n=18, 30%) that AI was more effective. Nevertheless, most of the respondents (n=51, 90%) agreed or strongly agreed that medical education learning specialists need to develop new skills to incorporate AI into their practices effectively, and about 40% (n=24) felt adequately prepared to adapt to changes in their job brought about by AI. Please see Figure 1 for medical education learning specialists' perspectives. 

Participants were generally positive in their perspectives toward the integration of AI, as indicated by descriptive statistics in Table 2. 

**Table 2:** Descriptive statistics for perspectives on the integration and impact of AI in medical education (N=61). Likert scale responses range from 1 (Strongly Agree) to 5 (Strongly Disagree); thus, reverse ordered for some of the questions to avoid survey bias. Data has been transformed with higher values indicating more positive perspectives on the impact of AI in descriptive statistics calculation.

Question	N	Mean	Median	Standard deviation
AI technology can significantly improve the personalization of medical education.	61	3.38	3	0.84
AI-driven analytics are more effective than traditional methods in identifying areas where medical students need improvement.	61	2.9	3	0.81
Medical education learning specialists need to develop new skills to effectively incorporate AI into their practices.	61	4.26	4	0.73
Medical education learning specialists will continue to play a critical role in medical education despite advances in AI.	61	4.64	5	0.78
The adoption of AI in medical education will widen the digital divide between different institutions and students.	61	2.67	3	0.89
The integration of AI tools in medical education enhances the learning experience for students.	61	3.75	4	0.79

Challenges and concerns about job security

Medical education learning specialists indicated that their top three challenges in their job were 1) learner hesitancy in using their services (n=45, 73.77%), 2) managing diverse learning needs and styles (n=32, 52.46%), and 3) keeping up with the latest technology (n=32, 52.46%).

Despite those challenges, medical education learning specialists were not concerned about job security. Please see Table 2 for frequency counts of their responses to questions regarding concerns.

**Table 3:** Frequency for concerns about job security caused by AI in medical education (N=61). Response Options (1=Strongly Agree, 2= Agree, 3= Neutral,  4=Disagree, 5=Strongly Disagree). Data are presented as numbers (frequency counts and % in 61 participants).

Questions	Strongly Agree	%	Agree	%	Neutral	%	Disagree	%	Strongly Disagree	%
AI advancements will primarily assist rather than replace human educators in the medical field.	19	31%	33	54%	4	7%	4	7%	1	2%
I am confident that my expertise and skills will remain valuable even as AI becomes more prevalent in medical education.	24	39%	30	49%	4	7%	1	2%	2	3%
I believe that AI will replace many tasks currently performed by medical education learning specialists.	1	2%	3	5%	9	15%	38	62%	10	16%
I feel adequately prepared to adapt to changes in my job role due to the adoption of AI technologies.	3	5%	20	33%	13	21%	21	34%	4	7%
The implementation of AI in medical education makes me worried about the future security of my job.	2	3%	7	11%	5	8%	33	54%	14	23%

**Figure 2:**
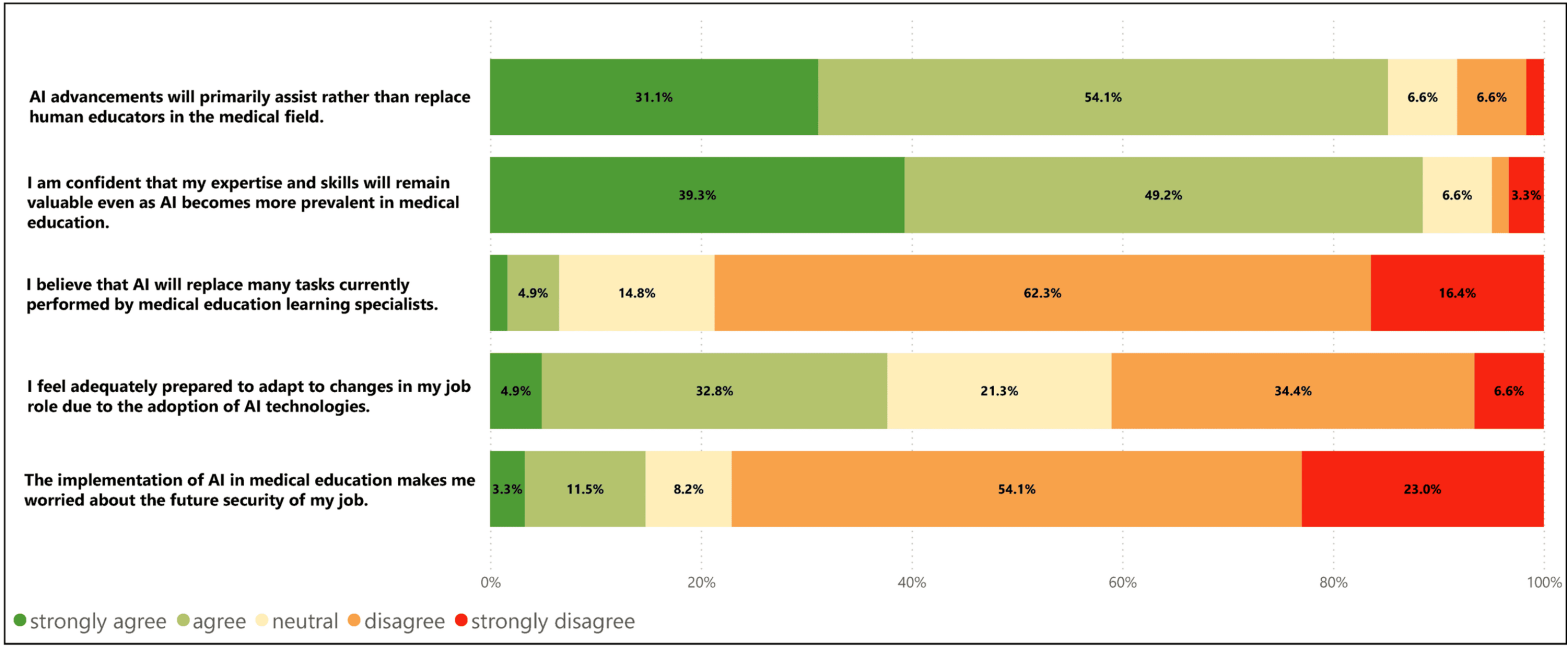
Challenges and concerns about job security caused by AI in medical education (N=61).

Less than 10% (n=6) felt that AI would replace the tasks they perform. Respondents were confident that the technological advancements would assist and not replace human educators (n=52, 85%), and about 90% (n=55) were confident that their expertise and skills would remain valuable even as AI becomes more prevalent (Figure 2).

Participants were generally not too concerned about the integration of AI, as indicated by descriptive statistics in Table 4. 

**Table 4:** Descriptive statistics for concerns about job security caused by AI in medical education (N=61). Likert scale responses range from 1 (Strongly Agree) to 5 (Strongly Disagree); thus, reverse ordered for some of the questions to avoid survey bias. Data has been transformed with higher values indicating less concern about job security caused by AI in descriptive statistics calculations.

Questions	N	Mean	Median	Standard deviation
AI advancements will primarily assist rather than replace human educators in the medical field.	61	4.07	4	0.89
I am confident that my expertise and skills will remain valuable even as AI becomes more prevalent in medical education.	61	4.2	4	0.89
I believe that AI will replace many tasks currently performed by medical education learning specialists.	61	3.87	4	0.81
I feel adequately prepared to adapt to changes in my job role due to the adoption of AI technologies.	61	2.95	3	1.07
The implementation of AI in medical education makes me worried about the future security of my job.	61	3.82	4	1.02

To explore broader patterns in perspectives, we created two composite scores: AI Optimism and AI Anxiety. AI Optimism was created based on agreement with the following statements: 1) The integration of AI tools in medical education enhances the learning experience for students, 2) AI technology can significantly improve the personalization of medical education, and 3) AI advancements will primarily assist rather than replace human educators in the medical field. AI Anxiety was created based on agreement with the following statements: 1)The implementation of AI in medical education makes me worried about the future security of my job, 2) I believe that AI will replace many tasks currently performed by medical education learning specialists, and 3) The adoption of AI in medical education will widen the digital divide between different institutions and students.

Using ANOVA tests, we analyzed AI Optimism and AI Anxiety scores across two key demographic groups: 1)Tech Proficiency and 2) AI Tool Adoption. 

It was found that there were no statistically significant differences in AI Optimism or AI Anxiety scores across tech proficiency levels or AI tool adoption groups (Table 5). This suggests that perspectives toward AI, both positive and anxious, are relatively consistent regardless of respondents’ technological skill level or whether they currently use AI tools.

**Table 5:** ANOVA test results for the composite scores (N=61).

	F-Statistic	p-Value
AI Optimism by Tech Proficiency	0.4526	0.7165
AI Anxiety by Tech Proficiency	0.9108	0.4416
AI Optimism by AI Tool Adoption	2.3247	0.1327
AI Anxiety by AI Tool Adoption	0.4892	0.4870

Tools used by medical education learning specialists

At the time of this survey, half of the respondents (n=31, 50.8%) had used AI tools in their practice, while the other half had not. The researchers read participants' narrative responses regarding the tools they have adopted and highlighted comment references (i.e., word(s), phrase(s), or sentence(s)) that described each of the AI tools they mentioned to capture the main ideas regarding how the participants used them. Data were coded using the constant comparative method. Based on a read of the data, emergent codes were identified, defined, and refined in an iterative process and then clustered into categories (e.g., which AI tool was used, how the tool was used, etc.). Two of the researchers independently coded the data and then met to compare and align their codings using the joint probability of agreement. The tools used by respondents are listed in Table 6.

**Table 6:** AI tools applicable for use by medical education learning specialists (n=25). Of the 61 participants, 25 provided valid responses to the open-ended question regarding AI tools adopted. ADA: The Americans with Disabilities Act; ADHD: Attention-deficit/hyperactivity disorder; AMBOSS: A medical knowledge platform that offers thousands of medical explanations and multimedia for free to help medical students and physicians learn efficiently and seamlessly; USMLE: The United States Medical Licensing Examination.

Tool	Website	Potential uses in medical education	References
ChatGPT	https://chatgpt.com/	To generate educational content or study materials, including class note summaries, practice questions, case studies, and explanations, quickly and efficiently. To create study schedules for professional examinations.	19
Claude	https://claude.ai/	To find and summarize literature on a research topic, generate/organize a literature review, design a research study and instruments, and suggest data analysis methods.	3
MS Copilot	https://copilot.microsoft.com/	To generate educational content or study materials, including class note summaries, practice questions, case studies, and explanations, quickly and efficiently. To create study schedules for professional examinations.	3
Goblin tools	https://goblin.tools/	AI-powered tools to help neurodiversity students, such as those with ADHD, to complete tasks, e.g., to break down a task, formalize language, and estimate time for completing a task, which is otherwise overwhelming to them.	2
Google Gemini	https://gemini.google.com/	To generate educational content or study materials, including class note summaries, practice questions, case studies, and explanations, quickly and efficiently. To create study schedules for professional examinations.	2
NotebookLM	https://notebooklm.google.com/	To create review materials, organization charts, or podcasts for students with the relevant content already cleaned up and filtered for accuracy by students.	2
Otter.ai	https://otter.ai/	To be used for ADA purposes for hearing or vision-impaired students, to automatically take meeting notes, make transcripts, and summaries. Can be used to prepare action items.	2
AMBOSS with a ChatGPT plug-in	https://www.amboss.com/us/gpt	To ask questions through a connection between ChatGPT and AMBOSS. To receive more accurate, up-to-date medical responses and a quick reference to AMBOSS articles in ChatGPT (need a login to AMBOSS to access articles)	1
Audiopen.ai	https://audiopen.ai/	To transfer audio messages into text notes and to be able to share with others. To make summaries.	1
Brainly	https://brainly.com/	An AI-powered learning companion for any subject, including medicine.	1
ChatGPT USMLE	https://chatgpt.com/g/g-pbeJ2CiUH-usmle-step-1-study-buddy	ChatGPT has a “USMLE Step 1 Study Buddy”.	1
Gamma - (Free)	https://gamma.com.ai/	AI Presentation Maker.	1
Jenni.ai	https://jenni.ai/	AI-powered research writing and citation assistance.	1

During the survey, participants were asked, “Have you adopted any AI tools in your practice? If yes, please share the tools you have adopted.” Some quotes from the narrative responses can illustrate how learning specialists were using AI tools specifically:

“I have utilized ChatGPT for a variety of things in the past few months but mainly I use it to fine tune [sic] wording in presentations or workshops that I am giving to students. I've also used it to find examples of case studies to incorporate into my workshops since I don't have a medical background. Another thing I have used it for is to help explain difficult medical concepts to better understand [sic] questions that my students are working on.” (Participant #15)

“Very simply, chatGPT. Share as an option for student who are [sic] struggling to find practice questions for a specific topic. We encourage students to confirm the reliability [sic] of information if there is a question, but overall, it has been useful.” (Participant #44)

Some other AI tools mentioned by the participants were Zoom AI companions for meeting notetaking or summary, Quizlet Plus or Memo (formerly known as PDF to Anki) for making flashcards, Osmosis or Blueprint (formerly Cram Fighter) for generating schedules. Learning specialists also suggested we should encourage students to take advantage of third-party resources that incorporate AI, but vet the results before relying on any AI output.

## Discussion

Medical education learning specialists' perspectives

In an age where artificial intelligence (AI) is rapidly transforming the educational landscape, the role of learning specialists has never been more critical. The findings suggest that a majority of respondents (n=57, 93%) agreed or strongly agreed that medical education learning specialists will continue to be valuable in medical education despite advances in AI technologies. Respondents also indicated that the top three challenges in their job were learner hesitancy in using their services (n=45, 73.77%), managing diverse learning needs and styles (n=32, 52.46%), and keeping up with the latest technology(n=32, 52.46%), and AI was not mentioned as a challenge at all.

Learning Specialists who came to the MELS conference round-table discussion were seeking guidance on how to use AI or how to give suggestions to students regarding using AI. Some of them shared that their students are afraid of using AI, terrified of being accused of plagiarism, and expressed concern about the future of their jobs due to the rapid advancement of AI and AI robotics. Some learning specialists shared stories and fond memories regarding their students, and agreed that most students still feel a stigma about utilizing a Learning Specialist, as echoed by our survey results, which indicated that learner hesitancy in using their services (n=45, 73.77%) was the top challenge in their job. Learning specialists still need to teach students that academic help-seeking is a healthy study skill, part of the self-regulated learning process.

We argue that the AI revolution is here to stay, and we must adapt to it. We must consider how we can live with and use AI for our teaching and learning benefits [14]. The importance of face-to-face interaction and the development of soft skills in medical education cannot be overstated [20]. Learning specialists bring a human touch to healthcare education, offering empathy, mentorship, and personalized feedback that AI cannot replicate, and learning specialists maintain that while AI can enhance learning, it lacks the nuanced understanding and critical thinking skills that human educators provide [21]. Students seek assistance for validation, resources, and accountability through human learning specialists who provide guided support through human interaction and relationships. Typically, a student’s changes in or implementation of learning strategies in follow-up steps are not predictable, and only human learning specialists can make the most appropriate and timely adjustments to customize the assistance we provide to help each student. AI can be used as a supplement when human learning specialists or tutors are not available [13], or when tasks are highly repetitive, e.g., answering test questions repeatedly for memorization, summarizing class notes, or creating study schedules. This may be one of the reasons why our survey respondents were confident that the technological advancements would assist and not replace human educators (n=52, 85%).

We may not want to rely solely on AI, especially for more complex knowledge and skills, or critical life-or-death decision-making [8,13]. It was found that AI technologies may encourage learners' dependence on technology and potentially cause metacognitive "laziness", which can affect their ability to self-regulate and engage deeply in learning [22]. Learning Specialists can find human tutors (as models) who have faced challenges during their academic journey to share their experiences, particularly how they overcame these challenges and achieved success, so that other students can learn from them. This might not be possible for AI tools or robots to implement and could be another reason why less than 10% (n=6) of respondents felt that AI would replace the tasks they perform.

AI systems can sometimes perpetuate biases from their training data, making human oversight critical to avoid AI hallucination, which occurs when an AI tool generates incorrect or misleading information that it presents as fact or truth. AI tutorials, writing tools, or methods will have to be tested or cross-examined to verify the accuracy of information [14], especially for critical medical treatment needs. Just as our study participants suggested, learning specialists need to teach healthcare education students to "vet the results before relying on any AI output."

When using AI for academic writing or references, we need to teach students to follow the institution’s guidelines for using AI, e.g., citing the use of ChatGPT for writing and integrating AI responsibly and ethically. Learning specialists can assume a portion of the responsibilities for teaching students the best practices of using AI for learning. As Zhang, Cai, Lee, et al. [21] have suggested, healthcare educators might need to maintain a balance between AI and educator-led teaching since medical students need to be trained to think independently and critically. In summary, we would like to echo what these other authors proposed through their writing:

“At present, AI’s lack of empathy and emotional intelligence makes human coaches irreplaceable” [10].

Tools used by medical education learning specialists

AI has been used to make life easier and to transform education. The AI tools reported by MELS survey respondents include: Chat GPT, Google Gemini, MS CoPilot, Audiopen.ai, NotebookLM, and Gamma - (Free), which are mainly used for generating educational content, study materials or schedules; Claude and Jennie.ai, which are used for research purposes; Otter.ai and Goblin tools, which are mainly for helping ADA (the Americans with Disability Act) accommodation or neurodiversity students; Brainly for learning companion, and AMBOSS with a ChatGPT plugin and ChatGPT USMLE (United States Medical Licensing Examination), which are specifically for medical professional exam preparation. These reported uses of AI applications are generally consistent with what Alkhaldi et al.’s study found from students, such as using ChatGPT to help complete written assessments and, to some extent, in their clinical work in medical school; while more students planned to use ChatGPT for investigating new medical topics, research, and exam preparation during residency [15].

Generative AI is considered most capable of conducting research efficiently, task automation, and enhancing content accessibility [23]. AI can process vast amounts of data, such as students’ test results, quickly, offering insights and recommendations based on patterns that might not be immediately obvious to human educators [24]. AI can assist in creating personalized learning pathways, providing instant feedback, and identifying areas where students need more support [25-26]. 

AI tools can be used to increase learning specialists’ speed and efficiency. However, the learning specialists at the MELS round-table discussion also cautioned against the development of AI into the efficiency of those scary robots in the movies. We want to avoid cultivating our medical students into those robotic doctors or nurses who lack attention and detail to human patients or who only operate according to an automatic checklist. Learning specialists also wanted to give healthcare education students a warning of overreliance on AI, which can cause metacognitive "laziness" and affect the depth of their learning and retention of knowledge and skills [22]. 

Both AI systems and human educators should continuously evolve to meet the changing demands of medical or other healthcare education [24]. Learning specialists can combine AI tools with traditional individualized teaching methods, including redesigning assessments based on AI integration [27], to create a more dynamic and effective learning environment to not only impart knowledge but also mentor human empathy. It’s imperative to train learning specialists to effectively use AI tools, ensuring they can integrate these technologies into their teaching strategies [28]. This need is also illustrated by our survey results, which indicated most of the respondents (n=51, 90%) agreed or strongly agreed that medical education learning specialists need to develop new skills to incorporate AI into their practices effectively, and about 40% (n=24) felt adequately prepared to adapt to changes in their job brought about by AI. There is a call for educators to take action to incorporate faculty development in AI across healthcare professions. Otherwise, we might risk creating a healthcare workforce unprepared to embrace the promise of AI [29].

Implications and future research

Medical education learning specialists must be knowledgeable about the changes in the AI landscape and how they will impact medical education. It was outside the scope of the study to understand the extent to which medical education learning specialists are trained or prepared to use AI in academic coaching. Future research should focus on examining learning specialists’ AI literacy first, and then providing specific preparation and training for them to integrate AI into academic coaching successfully. Training to learning specialists or educators can include workshops or online modules on topics, such as prompt engineering to achieve more effective and relevant AI responses, citations for AI use or creation, ethical and responsible AI use, evaluating AI tools, AI literacy, societal and cognitive Pros and Cons of using AI, and redesigning assessments based on AI integration [22,26-27]. For example, training can be offered on these topics:

Personalized learning: AI can analyze learning data, identify learning gaps, and recommend interventions. Learning specialists can learn to accelerate the process of getting help to students by using data provided by the specific AI tools used at their institution. Learning specialists can utilize test results, such as USMLE practice tests, and recommendations generated by AI based on students' strengths and weaknesses, and then make adjustments to customize each learner's strategies, pathways, and timeline, specifically, at a faster pace.

Redefining the role of the learning specialist: Learning specialists can learn to integrate AI to facilitate repetitive tasks or use AI to generate teaching strategies, which constitutes a human-AI collaboration [22], to improve the efficiency and effectiveness of their daily job responsibilities.

Increasing accessibility: Learning specialists can learn to provide students with 24-hour access to an AI-enabled intelligent assistant or chatbots when learning specialists or tutors are not available [26].

Since healthcare knowledge and skills require deep learning at such a fast pace, it is not sufficient to rely only on the surface memorization provided by most AI apps. It does require memorization to reach automaticity in healthcare daily professional practice; however, it demands a much higher level of metacognition and self-regulation [30] of the learner to achieve deep learning of the medical content. Human learning specialists or coaches can help students find the connections between the pieces of knowledge or skills to make diagnoses, to choose treatment plans, and to communicate with patients with high efficiency and warm human compassion. Future research should test the efficacy of this collaborative approach between learning specialists and AI tools for training healthcare education students. Future studies can also investigate the perspectives of medical or healthcare education students regarding their comparison between the use of AI tools and the medical education learning specialists for academic support.

Limitations

Several limitations warrant discussion regarding this research study. The first limitation is that the selected sample size may have impacted findings due to time constraints. More time may have allowed for more participants from the listserv to participate. A second limitation is that only learning specialists on the MELS listserv were invited to participate. The sample was drawn solely from U.S.-based MELS members, which limits the generalizability of findings to international contexts. A larger sample may have provided a broader viewpoint about AI in medical or other healthcare education. A third limitation of the study is that perspectives on AI have a cross-sectional nature, as perceptions on AI are rapidly evolving, e.g., pros and cons of cognitive impact from AI use. Only recently, it has been found that integrating AI does reduce the cognitive load for completing tasks; however, students’ learning using AI is on a shallower level and may not be retained as long [22,31]. We need to caution users of AI tools about this.

## Conclusions

Learning specialists generally agree that while they welcome training to use and integrate AI better, the technology is more likely to augment rather than replace their roles. A few reasons for their conclusion are: 1) The value of human interaction in education, especially in fields like medicine or healthcare, is irreplaceable. Learning specialists provide mentorship, emotional support, and nuanced feedback that AI cannot replicate; 2) AI can be used as a support tool to handle repetitive tasks, data analysis, and personalized learning plans, allowing learning specialists to focus on more complex and interpersonal aspects of teaching; 3) the role of learning specialists may evolve to include guiding students’ use of AI tools, interpreting AI-generated data, and integrating these insights into their teaching strategies; and 4) there is a continuous need for the expertise and critical thinking skills of learning specialists. These expertise and skills are vital for developing and refining educational content and methods for medical or healthcare education, something AI alone cannot achieve.

In summary, while AI will change the landscape of healthcare education, it is expected to work alongside learning specialists, enhancing their capabilities rather than replacing them. This collaborative approach can lead to more effective and personalized healthcare education.

## Appendices

**Table 7:** Medical learning specialist perspectives on AI questionnaire.

	Strongly agree	Agree	Neutral	Disagree	Strongly disagree
The integration of AI tools in medical education enhances the learning experience for students.	☐	☐	☐	☐	☐
Medical education learning specialists need to develop new skills to effectively incorporate AI into their practices.	☐	☐	☐	☐	☐
AI technology can significantly improve the personalization of medical education.	☐	☐	☐	☐	☐
Medical education learning specialists will continue to play a critical role in medical education despite advances in AI.	☐	☐	☐	☐	☐
AI-driven analytics are more effective than traditional methods in identifying areas where medical students need improvement.	☐	☐	☐	☐	☐
The adoption of AI in medical education will widen the digital divide between different institutions and students.	☐	☐	☐	☐	☐
The implementation of AI in medical education makes me worried about the future security of my job.	☐	☐	☐	☐	☐
I believe that AI will replace many tasks currently performed by medical education learning specialists.	☐	☐	☐	☐	☐
I feel adequately prepared to adapt to changes in my job role due to the adoption of AI technologies.	☐	☐	☐	☐	☐
I am confident that my expertise and skills will remain valuable even as AI becomes more prevalent in medical education.	☐	☐	☐	☐	☐
AI advancements will primarily assist rather than replace human educators in the medical field.	☐	☐	☐	☐	☐
